# Prevalence of Vitamin B12 Deficiency in Patients With Type 2 Diabetes Mellitus on Metformin Therapy: A Cross-Sectional Study

**DOI:** 10.7759/cureus.72184

**Published:** 2024-10-23

**Authors:** Shoaib Asghar, Haider Tanvir, Asad Riaz, Muhammad Hamza Ejaz, Mamuna Akram, Al Muktadir Chowdhury Evan, Salman Shahid

**Affiliations:** 1 Internal Medicine, Sheikh Zayed Medical College/Hospital Rahim Yar Khan, Rahim Yar Khan, PAK; 2 Internal Medicine, City Hospital, Multan, Multan, PAK; 3 Medicine, East Kent Hospital University Foundation Trust, Ashford, GBR; 4 Obstetrics and Gynecology, Lincoln County Hospital, United Lincolnshire Hospitals NHS Trust (ULHT), Lincoln, GBR; 5 Internal Medicine, Arrowe Park Hospital, Wirral, GBR; 6 Medicine, Bedfordshire Hospitals NHS Foundation Trust, Bedford, GBR

**Keywords:** diabetes mellitus management, duration of metformin therapy, metformin, metformin dose, metformin side effects, screening, serum vitamin b12, type 2 diabetes mellitus(t2dm), vitamin b12 deficiency, vitamin b12 deficiency anemia

## Abstract

Background

Metformin is frequently prescribed as a first-line oral hypoglycemic drug to treat insulin resistance-causing type 2 diabetes mellitus (T2DM). Long-term metformin use results in vitamin B12 deficiency, which is frequently overlooked and undiagnosed. A severe deficit may cause severe anemia and gastrointestinal, or neurological issues. Studies are scarce on this issue in Pakistani patients with T2DM.

The current study aimed to estimate the prevalence of metformin-induced vitamin B12 deficiency in T2DM patients and to explore how it relates to metformin dosage or duration of therapy.

Methodology

A descriptive cross-sectional study was conducted on 260 T2DM patients using metformin therapy for more than a year and attending the outpatient diabetes clinic and the medicine department of Sheikh Zayed Hospital, Rahim Yar Khan, Pakistan, from August 2022 to October 2023. All socio-demographic, clinical, and general characteristics were collected. Blood samples were taken to measure the serum vitamin B12 levels, and based on these levels, deficient and normal group characteristics were compared. Statistical analysis was performed using IBM SPSS Statistics for Windows, Version 23 (Released 2015; IBM Corp., Armonk, New York, United States).

Results

Based on the serum levels of vitamin B12 of the studied T2DM on the metformin regimen, the overall prevalence of vitamin B12 deficiency was found to be 36.54% (95). The B12 deficiency was higher among the age group of 41-50 years (109, 41.9%), female gender (150, 57.7%, p-value=0.0035), urban residents (194, 74.6%), non-smokers (197, 75.8%), and with a history of chronic disease (131, 50.4%). There was a statistically significant difference in vitamin B12 levels based on T2DM duration (p=0.012), with a higher prevalence in patients with a longer diabetes history of more than two years. There was no discernible statistical relationship between patients receiving different dosages of metformin (odds ratio (OR)=0.8627; 95% confidence interval (CI) (0.5195, 1.4326); p-value=0.568), durations of metformin (OR=0.7400; 95% CI (0.442, 1.2325); p-value=0.247), or intake of vitamin B12 (OR=0.8532; 95% CI (0.5073, 1.4351); p-value=0.549).

Conclusion

The prevalence of vitamin B12 deficiency impacted more than one-third of T2DM patients using metformin (36.54%). The risk of vitamin B12 deficiency may increase in females with higher metformin dosages and longer durations of treatment. Furthermore, a statistically significant correlation exists between vitamin B12 deficiency and the longer duration of T2DM.

These findings highlight the relevance of routinely monitoring serum levels of vitamin B12 among those with T2DM, especially when metformin is being given for over a year or at doses greater than 1000 mg per day. These preventive strategies will aid in the early detection of vitamin B12 deficiency, allowing patients to be treated with supplementation before problems such as anemia or neuropathies arise, resulting in improved quality of life and a lower socioeconomic burden.

## Introduction

Insulin resistance and corresponding insulin insufficiency are the hallmarks of type 2 diabetes mellitus (T2DM), a metabolic syndrome that manifests as hyperglycemia [1]. Biguanides (such as metformin) are still the primary oral hypoglycemic agents used to treat T2DM along with healthy lifestyle modifications [2]. Metformin can be used alone or in conjunction with other oral hypoglycemic drugs to reduce insulin resistance by increasing the sensitivity of insulin [2,3]. Metformin's adverse effects begin with the first dose and vary by dosage or frequency of use [3]. One of the most prevalent side effects of metformin treatment is gastrointestinal upset; nevertheless, vitamin B12 insufficiency is often overlooked [3,4].

Vitamin B12 is a water-soluble vitamin found in animal foods such as meat, fish, chicken, eggs, and dairy products [5]. The suggested dietary allowance for this vitamin is 2.4 micrograms per day [3-5]. It is essential for hematopoiesis, DNA synthesis, and neurological functions, and thus deficiency of vitamin B12 can result in megaloblastic anemia, gastrointestinal problems, and neurological impairments, including peripheral neuropathy [6-8].

Several studies have indicated a link between metformin use and a decrease in serum levels of vitamin B12 [7-12]. Numerous observational studies revealed that using metformin at greater doses or for longer periods of time, together with concurrent usage of proton pump inhibitors (PPIs) or histamine H2-antagonists, increased the risk of developing a vitamin B12 deficiency [9,10]. In 2016, a systematic study found that T2DM patients using metformin had significantly lower vitamin B12 levels compared to those not using it [10].

Metformin medication has been shown in studies to reduce serum vitamin B12 levels by 14% to 30% [11-13]. The high prevalence of T2DM and complications linked to a lack of vitamin B12 post-metformin treatment, as well as a dearth of studies from Pakistan addressing the relationship between the two, highlight a gap in our understanding of the issue.

The current study sought to assess the prevalence of metformin-induced vitamin B12 deficiency in T2DM patients and to explore how it relates to metformin dosage or duration of therapy.

## Materials and methods

Operational definition

According to the National Institute of Health USA [2] and Ralph G and Joshua WM (2022) [3]: Vitamin B12 deficiency (or cobalamin deficiency) is a medical condition in which the serum levels of vitamin B12 are less than 200 pg/mL, with levels up to 243 pg/mL being subclinically deficient (normal ranges 243-843 pg/mL).

The normal recommended dietary intake of vitamin B12 is 2.4 micrograms a day for both male and female adults (19+ years) [3]. And increased dietary allowance of 2.6 mcg during pregnancy and lactation [3].

Study design

Researchers conducted a descriptive cross-sectional study design involving patients on metformin therapy for T2DM who attended the medicine department and outpatient diabetes clinic of Sheikh Zayed Hospital, Rahim Yar Khan, Pakistan, between August 2022 and October 2023. A total of 260 patients were selected using a simple random sampling technique and screened to estimate the prevalence of vitamin B12 deficiency.

Sample size calculation

A mini-systematic review of 21 articles by Michael Atkinson et al. [10] found the prevalence of vitamin B12 deficiency between 20% and 30% among T2DM using metformin. The sample size was computed using the formula n = (Z^2 * P * (1-P)) / d^2. Where n is the calculated sample size, Z-score with a 95% confidence level of approximately 1.96, P is the 22% expected proportion in the population based on previous studies, and d is the precision/absolute error at 5% type 1 error (P<0.05). The final sample size was computed and rounded to 260.

Selection criteria

All T2DM patients older than 30 years and using metformin for more than a year were included in the study. This study excluded diabetes patients who were newly diagnosed, had a disease duration of less than one year, were on insulin therapy, were using proton pump inhibitors or H2 receptor blockers, were under the age of 30, were pregnant, had secondary diabetes or type 1 diabetes mellitus (T1DM), were alcohol consumers, suffered from inflammatory bowel disease, had previous gastrectomy or bariatric surgery, as well as patients with incomplete medical records, surgical histories, and other concomitant comorbidities. The exclusion criteria included individuals who were now or previously receiving multivitamin supplements or oral and intramuscular vitamin B12.

Data collection

Informed consent was obtained from participants and was approved by the ethical research board of Sheikh Zayed Medical College and Hospital. Interviews were conducted with all patients using a printed, tailored questionnaire proforma. The proforma included information about socio-demographic characteristics, weight, height, smoking history, duration of diabetes, duration and dose of metformin, use of insulin, possible comorbidities, and surgeries. An Excel spreadsheet was utilized to determine the daily intake of vitamin B12 based on food items ingested, and a validated and reliable food frequency questionnaire (FFQ) was employed to estimate dietary vitamin B12 intake (Appendix 1) [11]. As the main outcome, a blood sample was sent to the lab for measurement of vitamin B12 levels. Patients were categorized as having normal or vitamin B12 deficiency based on these values, and the clinical characteristics of the two groups were compared.

Statistical analysis

IBM SPSS Statistics for Windows, Version 23 (Released 2015; IBM Corp., Armonk, New York, United States) was used for the statistical analysis. The categorical variables were measured in terms of frequencies and percentages. Descriptive statistics of continuous data were expressed in the form of mean ± standard deviation (SD). The study applied the student’s t-test for continuous variables and Pearson's chi-square test of independence for categorical data to compare the vitamin B12 deficient and normal groups. A logistic regression analysis was employed to explore vitamin B12 deficiency with respect to socio-demographics, clinical factors, and medications, and odds ratios (OR) were obtained. After calculating the p-value, a result of less than 0.05 was deemed significant.

## Results

A total of 260 T2DM patients aged between 30 and 64 years on metformin therapy were included in this study. The majority of respondents (109 (41.9%)) were aged 41 to 50 years, followed by 92 (35.4%) aged 31 to 40 years, and the remaining 59 (22.7%) were aged 51 to 64 years. The majority of respondents, 150 (57.7%), were female, and of the total respondents, 208 (80%) were married and 194 (74.6%) residing in urban areas. The largest group was non-smokers (75.8%), whereas 63 (24.2%) were smokers.

A comparison between normal and vitamin B12-deficient groups showed a significant difference for the female gender (p-value=0.0035). No statistically significant difference was noted for age, marital status, residence, or history of smoking. Table 1 displayed the frequency distribution with percentages for socio-demographic variables according to serum levels of vitamin B12. The values of the chi-square test and its corresponding p-value for each variable were also incorporated in the table.

**Table 1:** Distribution of the socio-demographic variables of studied type 2 diabetes mellitus patients on metformin according to serum levels of vitamin B12 (n=260) The chi-square test is applied.

Socio-Demographic Variables	Number (%) (N = 260)	Serum Levels of Vitamin B12 (n=260)			
		Vitamin B12 Deficiency (n1 = 95)	Normal Vitamin B12 (n2= 165)	Chi-Square Value	p-value
Age					
31-40 (years)	92 (35.4)	28 (29.5%)	64 (38.8%)	2.531	0.282
41-50 (years)	109 (41.9)	42 (44.2%)	67 (40.6%)		
51-64 (years)	59 (22.7)	25 (26.3%)	34 (20.6%)		
Gender					
Female	150 (57.7)	66 (69.5%)	84 (50.9%)	8.512	0.0035
Male	110 (42.3)	29 (30.5%)	81 (49.1%)		
Marital status					
Married	208 (80)	74 (77.9%)	134 (81.2%)	0.415	0.520
Single	52 (20)	21 (22.1%)	31 (18.8%)		
Residence					
Urban	194 (74.6)	65 (68.4%)	129 (78.2%)	3.033	0.082
Rural	66 (25.4)	30 (31.6%)	36 (21.8%)		
Smoking					
Yes	63 (24.2)	22 (23.2%)	41 (24.8%)	0.094	0.759
No	197 (75.8)	73 (76.8%)	124 (75.2%)		

The mean and standard deviation of serum vitamin B12 levels were 159.8 ± 54.68 pg/mL, with a range of 89-364 pg/mL among all studied T2DM patients on a metformin regimen. There were 95 (36.54%) patients with vitamin B12 deficiency (<200 pg/mL) and 165 (63.46%) patients with normal vitamin B12 levels. So, the prevalence of vitamin B12 deficiency among T2DM on the metformin regimen was 36.54% (95, n=260).

The mean and standard deviation for height and weight of T2DM participants under study were 163.54 ± 9.69 cm and 83.15 ± 19.35 kg, respectively. The group deficient in vitamin B12 received a dose of 1406.4 ± 358.2 mg of metformin, with an average length of usage of 2.13 ± 0.60 years. There was no discernible statistical relationship between the metformin therapy duration or dosage among the vitamin B12 groups. Patients with or without vitamin B12 deficiency reported using metformin for the same extended period. Table 2 displays the mean ± SD of the clinical and general variables/medications according to serum levels of vitamin B12.

**Table 2:** Descriptive characteristics of clinical & general variables/medications of studied type 2 diabetes mellitus patients on metformin according to serum levels of vitamin B12 (n=260) *Normal intake: The recommended dietary allowance for vitamin B12 is 2.4 micrograms a day for both male and female adults (19+ years) [3]. And 2.6mcg during pregnancy and lactation [3]. ** Chronic diseases (kidney, heart, and neurological diseases) P-values based on the t-test and chi-square test.

Clinical & General Variables/Medications	Number (%) / Mean ± SD (Total n = 260)	Serum Levels of Vitamin B12 (Total n=260)			
		Vitamin B12 Deficiency (n1= 95)	Normal Vitamin B12 (n2 = 165)	Chi-Square Value	p-value
Height					
Height (cm)	163.54 ± 9.69	164.39 ± 8.76	163.12 ± 9.56		0.289
Weight					
Weight (kg)	83.15 ± 19.35	87.95 ± 22.65	84.29 ± 18.68		0.161
Duration of metformin					
Metformin duration overall (years)	1.95 ± 0.78	2.13 ± 0.60	2.06 ± 0.67		
1-2 years	119 (45.8)	39 (41.1%)	80 (48.5%)	1.342	0.247
More than 2 years	141 (54.2)	56 (58.9%)	85 (51.5%)		
Dose of metformin					
Metformin dose overall (mg)	1268.1 ± 496.5	1406.4 ± 358.2	1369 ± 395.6		
≤ 1000 mg	121 (46.5)	42 (44.2%)	79 (47.9%)	0.326	0.568
≥ 1000 mg	139 (53.4)	53 (55.7%)	86 (52.1%)		
Intake of vitamin B12					
Low	92 (35.8)	35 (36.8%)	67 (70.6%)	0.358	0.549
Normal*	168 (64.6)	60 (63.2%)	98 (59.4%)		
Duration of type 2 diabetes mellitus (T2DM)					
1-2 years	97 (37.3)	26 (27.4%)	71 (43%)	6.323	0.012
More than 2 years	163 (62.7)	69 (72.7%)	94 (57%)		
Hypertension					
Yes	121 (46.5)	39 (41.1%)	82 (49.7%)	1.811	0.178
No	139 (53.5)	56 (58.9%)	83 (50.3%)		
Chronic diseases **					
Yes	131 (50.4)	51 (53.7%)	80 (48.5%)	0.652	0.419
No	129 (49.6)	44 (46.3%)	85 (51.5%)		

There was a statistically significant difference in vitamin B12 levels based on T2DM duration (p=0.012), with a higher prevalence of 62.7% (163) in patients with a longer history of more than two years. There was no evident association between serum vitamin B12 levels and the patient's dietary intake (p=0.549), history of hypertension (p=0.178), and other chronic diseases (p=0.419).

Table 3 presents the odd's ratio and p-values of potential risk factors for vitamin B12 deficiency among T2DM patients using metformin therapy, based on a logistic regression model.

**Table 3:** Potential risk factors for vitamin B12 deficiency among T2DM patients using metformin (n=260) OR: odd’s ratio; CI: confidence interval; T2DM: type 2 diabetes mellitus *Logistic regression is applied

Potential Risk Factors for Vitamin B12 Deficiency	Reference Group	OR (95% CI) *	p-value
Age	41-50 (years)	1.1591 (0.6958, 1.9310)	0.570
Gender	Male	2.1946 (1.2882, 3.7388)	0.0035
Height (cm)	Height (cm)	1.197 (0.6276, 2.0876)	0.289
Weight (kg)	Weight (kg)	2.604 (1.7873, 4.4673)	0.161
Marital status	Married	0.8152 (0.4375, 1.5191)	0.520
Residence	Urban	0.6047 (0.3423, 1.0680)	0.082
Smoking	Smoking - Yes	0.9115 (0.5036, 1.6496)	0.759
Duration of metformin	1-2 Years	0.7400 (0.442, 1.2325)	0.247
Dose of metformin	≥ 1000 mg	0.8627 (0.5195, 1.4326)	0.568
Intake of vitamin B12	Intake of vitamin B12 - Yes	0.8532 (0.5073, 1.4351)	0.549
Duration of T2DM	1-2 Years	2.00 (1.1608, 3.4614)	0.012
Hypertension	Hypertension - Yes	0.7049 (0.4232, 1.1741)	0.178
Chronic diseases	Chronic Diseases - Yes	1.2315 (0.7426, 2.0423)	0.419

The analysis showed that gender and duration of T2DM were substantially linked with vitamin B12 deficiency among T2DM patients using metformin. The odds ratio of gender indicated that female patients were more likely than males to be deficient in vitamin B12. The results also revealed that patients who had T2DM for more than two years had greater odds of having vitamin B12 deficiency compared to T2DM patients who had it for one to two years.

## Discussion

This study reported a high prevalence of vitamin B12 deficiency of 36.54% (serum levels less than 200 pg/mL) among T2DM subjects using higher doses of ≥ 1000 mg metformin for over a year. Many research studies found that the prevalence of metabolically verified vitamin B12 deficiency in patients taking metformin ranged from 4% to 51% [9-14]. The prevalence results of this study are consistent with the results reported by Iftikhar R et al. of Pakistan at 31% [12] and Yousef Khan F et al. of Qatar at 30.7% [13]. Shahwan M et al. of Ajman UAE [14] noted similar and higher results at 48% and Malla et al. of Nepal at 50.95% [15]. Whereas, lower prevalences were confirmed by Damião CP et al. at 22.5% [16], Chapman LE et al. of Brazil at 21.5% [17], and Almatrafi SB et al. of Saudi Arabia at 17.5% [11]. Methodological discrepancies, cutoff values used in vitamin B12 deficiency studies, and various dietary and cultural practices, which all play a role in vitamin B12 levels, could explain this apparent variation. Additional blood tests that are more accurate in diagnosing vitamin B12 deficiency include serum homocysteine and methylmalonic acid levels [13,15,16]. Nevertheless, our institution did not have availability of these tests.

Metformin is frequently prescribed as a first-line oral hypoglycemic drug of choice; slow up-titration of therapy is recommended to alleviate associated gastrointestinal side effects such as diarrhea, nausea, flatulence, indigestion, vomiting, and abdominal pain, with diarrhea and nausea being the most common [8]. A severe deficit of vitamin B12 due to any reason may cause severe anemia and further gastrointestinal or neurological problems [9-12]. This study found that the deficiency of vitamin B12 was more common in middle age, with a majority of 41-50-year-olds (44.2%), females (69.5%), married (77.9%), urban inhabitants (68.9%), and non-smokers (76.8%). Gender is found to be significantly associated with deficiency of vitamin B12 for the present study. These results are consistent with those reported by Almatrafi SB et al. [11] and Damião CP et al. [16]. The studies by Chapman LE et al. [17] and Tan WY et al. [18] supported that female sex [9-13,16] and age group 40-55 years were independent indicators of vitamin B12 deficiency in T2DM patients on metformin regimens for a longer duration of more than two years [16-18].

The mean and standard deviation for height and weight of T2DM participants under study were 163.54 ± 9.69 cm and 83.15 ± 19.35 kg, respectively. Taller (p-value=0.289) and overweight (p-value=0.161) people were more inclined to have low serum vitamin B12 levels, most likely due to higher requirements, as reported by Almatrafi SB et al. of Saudi Arabia [11], Hasan NU et al. of Pakistan [19], and Singh AK et al. of India [20].

The group deficient in vitamin B12 received a dose of 1406.4 ± 358.2 mg of metformin (p-value=0.448), with an average length of usage of 2.13 ± 0.60 years (p-value=0.247). Patients with or without vitamin B12 deficiency reported using metformin for the same extended time. For the deficient group, 41.1% (39, n=95) had a length of one to two years, and 58.9% (56, n=95) had a duration of more than two years of taking metformin. Similar findings were observed in local studies in Pakistan by Hasan NU et al. [19] and Miyan Z et al. [21], as well as international investigations by Beulens JW et al. [22], Gao L et al. [23], and Gogia S et al. [24], which found that longer duration of metformin and higher daily dosage were correlated with vitamin B12 deficiency, respectively. The study by Singh AK et al. [20], Miyan Z et al. [21], and Gogia S et al. [24] reported that the deficiency of vitamin B12 was associated with neuropathy in subjects on metformin for >two years and hence needed screening.

The studies by Al-Hamdi A et al. [25], DS Kalani et al. [26], and Aroda VR et al. [27] found that a greater dose of metformin and a longer duration of diabetes were substantially linked with vitamin B12 deficiency. Damião CP et al. [16] reported that the treatment of T2DM with metformin and concomitant use of PPI/H2-antagonists are associated with a higher chance of developing B12 deficiency. Whereas the Tan WY et al. study found a correlation between vitamin B12 deficiency and HbA1c, homocysteine levels, metformin dosage, and duration [18]. Hypertension (p-value=0.178) and other comorbidities (p-value=0.419) were not observed as potential risk factors in this study.

In terms of intake of vitamin B12, 35.8% (92, n=260) had a low intake, while 64.6% (168, n=260) had a normal intake. No significant correlation was found between serum vitamin B12 levels and dietary vitamin B12 intake of patients (p-value=0.709). Similar results were reported by Al Saeed RR et al. [9], Gao L et al. [23], and Khattab R et al. [28], who discovered that numerous dietary and cultural effects were the cause of insignificant statistics, but the majority of patients had a low dietary intake of vitamin B12, leading to around 44% higher risk of deficiency. Low dairy intake and some gastrointestinal diseases might be considered risk factors for having low vitamin B12 levels [28].

There was a significant statistical difference in vitamin B12 levels based on T2DM duration (p=0.012), with a higher prevalence in patients with a longer history of more than two years (62.7%, n=260) and vitamin B12 deficiency identified in 72.7% (69, n=95). These figures accorded with several studies [9,14-16,19, 23] as well as those published by Ahmed MA et al. [29] and Yazidi M et al. [30], which support the prolonged duration of diabetes mellitus as the possible culprit of the deficiency.

This research's core merit was how we accounted for several causal factors relevant to vitamin B12 deficiency and included statistics regarding metformin treatment. Also, we used a standardized FFQ to generate a dietary evaluation for vitamin B12 intake. However, the limitations of this study included a small data sample from a single hospital, a lack of baseline vitamin B12 level before metformin therapy, and did not utilize other more sensitive ways of evaluating deficiency indicators like serum homocysteine and methylmalonic acid levels since they were not available in the hospital. Furthermore, the study was restricted to diabetes patients under the age of 30, using insulin therapy, using proton pump inhibitors or H2 receptor blockers, consuming alcohol, having type 1 diabetes mellitus (T1DM) or secondary diabetes, suffering from inflammatory bowel disease, and having previously undergone gastrectomy or bariatric surgery.

## Conclusions

The prevalence of vitamin B12 deficiency impacted more than one-third of T2DM patients using metformin (36.54%). The risk of vitamin B12 deficiency may increase in females with higher metformin dosages and longer durations of treatment. Furthermore, a statistically significant correlation exists between vitamin B12 deficiency and the longer duration of T2DM.

These findings highlight the relevance of routinely monitoring serum levels of vitamin B12 among those with T2DM, especially when metformin is being given for over a year or at doses greater than 1000 mg per day. These preventive strategies will aid in the early detection of vitamin B12 deficiency, allowing patients to be treated with supplementation before problems such as anemia or neuropathies arise, resulting in improved quality of life and a lower socioeconomic burden.

## Appendices

Appendix 1

**Table 4:** Food frequency questionnaire for vitamin B12 (cobalamin) intake (commonly used food items, serving sizes, and frequencies)

Food Items	Average Serving Size	Serving Size (if not average)		How Often? (Complete One Column Only) -Enter Number in that column-				Content of Vitamin B12 per Serving (µg) (Leave Blank)
		Small (Tick)	Large (Tick)	Daily	Weekly	Monthly	Never Or Rarely	
Dairy and dairy products								
Milk								
Curd cow or yogurts								
Butter/cream								
Processed cheese								
Paneer (cottage cheese)								
Egg (hen, whole)								
Meats and fish products								
Chicken								
Goat								
Lamb								
Beef								
Organ red meat - liver								
Organ red meat - kidney								
Lamb brain								
Fish or fish products								
Sushi								
Other seafoods (clams, oysters, prawns, mussels, crabs, sardines)								
Vitamin B12 fortified foods or drinks								
Breakfast cereals (fortified)								
Any fortified food								
Any fortified drink								
Cooking oils								
Dried food items and others								
Nuts and seeds								
Beans, peas, or lentils								
Dried plums								
Soy products								
Dark chocolates								
Fruits and vegetables								
Banana								
Oranges								
Guava								
Mango								
Apple								
Kiwi								
Berries (blueberries, blackberries, mulberries)								
Beetroot								
Potato								
Spinach, broccoli, brussels sprouts, asparagus, parsley								
Mushrooms								
